# Preferred Treatment Patterns of Retinopathy of Prematurity: An International Survey

**DOI:** 10.3390/pediatric16030069

**Published:** 2024-09-13

**Authors:** Amy T. Wang, Shuan Dai

**Affiliations:** 1 Department of Ophthalmology, Queensland Children’s Hospital, South Brisbane 4101, Australia; amytwang@alumni.ubc.ca; 2 Menzies Health Institute Queensland, Griffith University, Gold Coast 4215, Australia

**Keywords:** retinopathy of prematurity, anti-vascular endothelial growth factor, laser photocoagulation, intravitreal injection

## Abstract

This paper assesses the preferred treatment patterns for retinopathy of prematurity (ROP) and examine trends in anti-vascular endothelial growth factor (VEGF) use for ROP. **Methods**: A retrospective survey consisting of 14 questions was distributed to paediatric ophthalmology interest groups internationally. Main outcome measures included treatment patterns, proportion of anti-VEGF use in different stages of ROP; and comparison of first-line treatments as well as repeat anti-VEGF treatments. **Results**: Fifty-four ophthalmologists from 11 different countries responded to the survey. The number of respondents per question, except one, ranged between 50–54. Per annum, there was an average number of 394 infants screened by each respondent. Anti-VEGF was the preferred treatment method for aggressive (A)-ROP (64.1%), Type 1 ROP in zone 1 (71.7%), and Type 1 ROP in posterior zone 2 (56.6%). The majority used laser as the first-line treatment of Type 1 ROP in anterior zone 2 (73.6%) and Type 1 ROP in zone 3 (79.2%). Laser was the preferred treatment modality utilised in infants requiring repeat treatment following anti-VEGF injection. The preferred anti-VEGF agent was bevacizumab administered at a dose of 0.625 mg. **Conclusions**: Anti-VEGF as first-line therapy has been increasing. Anti-VEGF appears to be the first-line treatment of choice for A-ROP, Type 1 ROP in zone 1 and posterior zone 2 and laser for Type 1 ROP in anterior zone 2 and zone 3.

## 1. Introduction

Retinopathy of prematurity (ROP), a vascular proliferative disease involving the upregulation of vascular endothelial growth factor (VEGF), is the leading cause of preventable blindness in infants born prematurely worldwide [1]. Cryotherapy, once the mainstay of treatment, has been superseded by laser photocoagulation in recent years [2]. Though retinal laser photocoagulation was considered the definitive treatment for ROP, recent advances in anti-VEGF therapies have gained momentum due to their direct role on the pathophysiology of ROP and therapeutic efficacy [3,4]. Despite several large, randomized control studies and multiple case studies, there appears to be no universal consensus in the literature concerning an indication of treatment, dosage, or preferred agents of anti-VEGF in ROP [5,6,7,8,9].

Large clinical trials of utilising anti-VEGF in pre-term infants have provided a framework for clinicians selecting the appropriate drug and dose in the past decade [8,9,10,11]. The BEAT-ROP study, the first randomized control trial involving multiple centres in the United States, assessed the efficacy of intravitreal bevacizumab as a monotherapy when compared to conventional laser therapy for the treatment of zone 1 or posterior zone 2 stage 3 with plus disease [8]. The study supported the use of anti-VEGF (bevacizumab) over laser for zone 1 disease [8]. An international randomized clinical trial (RAINBOW) compared ranibizumab at two different doses (0.1 mg, 0.2 mg) to laser, and although ranibizumab did not achieve statistical significance as superior to laser treatment, a benefit was shown with the 0.2 mg dose [9]. Based on the RAINBOW study results, ranibizumab was approved in the European Union and India for the treatment of ROP, whilst in many other countries, it remains for off-label use [9,12]. There is no consensus, internationally, of changes in the uptake of anti-VEGF following recent advances [12,13]. However, at the time of writing, Winter et al. demonstrated that there is a reversal of preference from laser to anti-VEGF in Germany [14]. This study aims to determine the current patterns of preferred treatment worldwide for ROP and the trends of anti-VEGF use following the RAINBOW study.

## 2. Materials and Methods

A structured questionnaire delivered through a secure online platform, SurveyMonkey, was distributed to paediatric ophthalmologists (including retinal specialists and International Committee for Retinopathy of Prematurity 3rd edition committee members) treating ROP worldwide via the Paediatric Ophthalmology International ListServ following ethics approval from the local research ethics board in June 2023. A retrospective survey of 14 multiple choice questions with prefilled options aimed to gain understanding from clinicians regarding the number of infants screened and treated per annum (Table 1). Clinicians were asked about their preferred treatment modality for Type 1 ROP. The uptake of intravitreal anti-VEGF as a first-line treatment was assessed between two timelines within a ten-year period (2012 to 2022): before and after 2019 (publication year of the RAINBOW study). Each clinician is encouraged through the survey to recall their treatment practices for Type 1 ROP during the two time frames previously mentioned. Clinicians answering the survey individually commented on their treatment practices between 2012 to 2022. Repeat treatment patterns following the initial failure of anti-VEGF therapy was also assessed. The responses were separated into subgroups based on the respondents’ respective countries and their corresponding income classification, with developed countries classified as higher income countries and middle to lower income countries classified as developing countries (according to the World Bank classifications of countries). Wilcoxon signed-rank tests were used to calculate the differences between first-line treatment and repeat treatment modalities before and after 2019, with statistical significance of *p* < 0.05.

## 3. Results

Fifty-four ophthalmologists from 11 different countries responded to the survey. Thirteen out of the total fourteen survey questions had response rates between 50 to 54. Thirty-five respondents (64%) were from developed countries: Australia, New Zealand, USA, Israel, and Canada. Nineteen respondents (36%) were from developing countries: China, Malaysia, South Africa, Mexico, Philippines, and Ukraine. The reported total number of infants screened for ROP per annum was 10,195 from clinicians practicing in developed countries and 9488 in developing countries. Respondents reported screening a mean of 394 infants per annum (range from 25 to 3000). Between 2012 to 2022, respondents reported a total of 6813 infants who had treatment for ROP. Of the total number of infants treated for ROP, 2997 (43.9%) received laser retinal photocoagulation, and 3816 (56.1%) received intravitreal anti-VEGF treatment. Of the total number of infants who received anti-VEGF between 2012 to 2022, 3581 (93.8% of total number of infants who received anti-VEGF) were treated as first-line therapy. 

### 3.1. First-Line Treatment 

Out of 53 respondents, anti-VEGF was preferred among clinicians treating posterior disease (A-ROP [64.1%], Type 1 ROP in zone 1 [71.7%], posterior zone 2 [56.6%]) and laser monotherapy preferred in anterior disease (Type 1 ROP in anterior zone 2 [73.6%], Type 1 ROP in zone 3 [79.2%]). A small subset of clinicians reported using a combination of laser and anti-VEGF, as well as cryotherapy monotherapy (Table 2).

**Figure 1:**
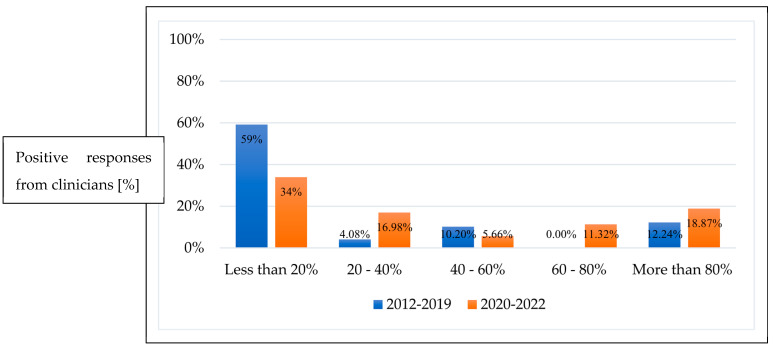
Percentage of ROP infants treated with anti-VEGF as first-line therapy between 2012–2019 and 2020–2022.

Prior to 2019, 32 clinicians (60.3%) did not prefer to treat ROP with anti-VEGF, reporting its use <20% of the time. Post 2019, there was global increased uptake of anti-VEGF as the first-line treatment. The percentage of clinicians who responded they would treat 20–40%, 60–80%, and more than 80% of infants with anti-VEGF as the initial therapy rose from 4 to 16.9%, 0 to 11.3%, and 12.2 to 18.8%, respectively (*p* < 0.001) (Figure 1). 

### 3.2. Repeat Treatment Post Initial Anti-VEGF Monotherapy 

**Figure 2:**
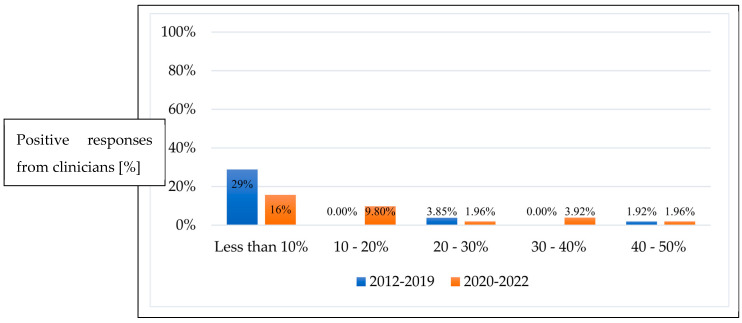
Percentage of ROP infants requiring repeat treatment with anti-VEGF between 2012–2019 and 2020–2022.

Prior to 2019, 44 clinicians (84.6%) preferred not to use anti-VEGF for re-treatment, reporting its use less than 10% of the time. A wide distribution of responses from clinicians was reported post-2019, with varied uptake in the increase of anti-VEGF utilisation for repeat treatment (Figure 2). The percentage of clinicians who responded that they would treat 10–20%, 30–40%, and 40–50% of infants with anti-VEGF as repeat treatment post initial anti-VEGF therapy failure increased from 0 to 9.8%, 0 to 3.9% and 1.9 to 2%, respectively (*p* = 0.06). 

Laser was the preferred modality for infants requiring repeat treatment for ROP after initial anti-VEGF treatment in both developing and developed countries. Respondents mainly used laser as repeat treatment for Type 1 ROP in anterior zone 2 (69% of the time) and Type 1 ROP in zone 3 (76% of the time). A subset of clinicians 13 (29%) reported using a combination of anti-VEGF and laser or anti-VEGF solely for repeat treatments in all stages. 

### 3.3. Preferred Anti-VEGF Agents

Most clinicians preferred bevacizumab (30 respondents; 60%), followed by ranibizumab (17 respondents; 34%). A small subset (three respondents; 6%) reported using aflibercept or a combination of three previously mentioned agents. Bevacizumab was preferred by respondents in developed countries (26 respondents, 81.2%), compared with developing countries (four respondents; 26.6%). A higher percentage of respondents from developing countries preferred ranibizumab (11 respondents, 73.3%) compared to clinicians from developed countries (six respondents; 18.7%).

The dosage preferred by most clinicians was bevacizumab at 0.625 mg (18 respondents; 60%) followed by bevacizumab at half dose 0.325 mg (12 respondents; 40%). A small subset of clinicians used ranibizumab at a 0.2 mg dose followed by 0.1 mg doses (ten, seven respondents; 58.9%, 41.1%, respectively).

### 3.4. Follow-Up Timeframe

The follow-up timeframes for infants who completed anti-VEGF therapy and prior to discharge from active screening were variable. Twenty-one respondents (38%) reported follow-up up to 60 weeks post-menstrual age, fifteen respondents (28%) at 55–60 weeks, and five respondents (10%) at 50–55 weeks. There was no consensus on follow-up timeframes post-discharge from active ROP screening, with 19 respondents (36%) reporting 3 months, 17 respondents (34%) reporting 3–6 months, and 10 respondents (20%) reporting between 2 months and 1 year. 

## 4. Discussion

The overall anti-VEGF trends in the survey correspond with two landmark trials, BEAT-ROP and RAINBOW, published in 2011 and 2019, respectively. Shifting away from laser photocoagulation for Type 1 ROP, clinicians reported anti-VEGF as the preferred modality of choice over laser for A-ROP and Type 1 ROP in zone 1 and posterior zone 2 [7]. This change in paradigm was driven by the results of the BEAT-ROP study, and its influence mirrors the clinician-reported use of anti-VEGF between 2012 and 2019 in this survey. From 2019 onwards, there is an increase in reported anti-VEGF use as a first-line treatment as opposed to laser for ROP, which coincides with the RAINBOW study. The popularity of anti-VEGF monotherapy could also be attributed to the significantly shorter time required to anaesthetize the infant, and ability to conduct treatment bedside without general anaesthesia without intubation [15]. This may be safer and easier in cases of infants with unstable systemic conditions [15].

Laser photocoagulation is still predominately used in the treatment of ROP localized in the peripheral retina (anterior zone 2 and zone 3), presumably due to its established regard as a gold standard treatment [1,16]. Laser photocoagulation is suggested to inhibit angiogenesis for longer, which leads to more time between treatment and retreatment [16]. Once vascularisation has reached the peripheral retina, the balance between expanding the visual field (facilitated by using anti-VEGF) and administering definitive treatment (via laser) tilts in favour of definitive treatment. This is consistent with respondents preferring laser as a modality of choice for repeat treatment following initial anti-VEGF monotherapy. 

The most reported anti-VEGF agent used was bevacizumab at two doses, 0.625 mg and 0.325 mg, followed by ranibizumab at 0.2 mg and 0.1 mg, respectively. This is consistent with large clinical trials and smaller studies published that suggest efficacy with both ranibizumab and bevacizumab at the half and quarter adult dosing [8,9]. Although clinicians have appeared to change their practice corresponding to the RAINBOW study, the majority have not adopted the drug it supported: ranibizumab. Cost, earlier recurrence of disease, and a higher rate of recurrence might be some of the reasons clinicians chose bevacizumab over ranibizumab [17]. 

An international trial reported treatment success with aflibercept 0.4 mg for active ROP, and another historical cohort study demonstrated efficacy of aflibercept in Type 1 ROP [11,18]. Despite this, there remains limited evidence in the literature on the usage of aflibercept in Type 1 ROP. This may be the reason why only a small subset of clinicians reported the use of aflibercept as a first-line treatment. 

There was high variation in reported follow-up timeframes post-completion of ROP treatment, prior to discharge from active ROP screening, with responses ranging between 50 and 60 weeks. This trend amongst clinicians may be influenced by the reports of mean time to recurrence of ROP in BEAT-ROP (19.2 weeks after bevacizumab injection) and RAINBOW (8 weeks after ranibizumab injection) trials [8,9]. A recent study reported completion of retinal vascularization post 0.625 mg bevacizumab at 38.18 ± 6.5 weeks and 23.86 ± 9.3 weeks post aflibercept administered at 1 mg [18]. There was no consensus amongst clinicians for follow-up post-discharge from active screening, as responses varied anywhere from 3 months to 12 months. Whilst late reactivation of ROP after receiving anti-VEGF monotherapy is not commonly reported in the literature, there is a case of an asymptomatic child that developed a late recurrence of ROP 6 years post treatment [19,20]. This further emphasizes the importance of long-term follow up. The optimal method for detecting recurrence of retinal neovascularization would be to utilise RetCam imaging which provides a photographic record, but indirect ophthalmoscopy examination by the clinician bedside is also utilised [21].

It is of interest that ranibizumab, a relatively high-cost medication, is the predominant choice of anti-VEGF in ROP treatment amongst clinicians from developing countries. Most clinicians from developed countries reported the use of bevacizumab, the lower cost agent, as compared with ranibizumab. Further studies may be recommended to explore this difference in health care resource expenditure and its economic impact. 

Whilst every effort was made in the disclaimer section of the survey that respondents should rely upon clinical data, there was no method to monitor the respondents’ reported data. Respondents, as such, are subject to elements of recall bias. Possibilities for future studies would be to collect retrospective data in a predesigned proforma from clinicians. 

This survey provides insight into current ROP treatment patterns internationally. The frequency of anti-VEGF use amongst clinicians is higher in the 2019 to 2022 period as compared with 2012 to 2018. Overall, more clinicians preferred anti-VEGF as the first-line treatment for posterior disease (A-ROP, Type 1 ROP in zone 1, posterior zone 2); and laser for anterior disease (Type 1 ROP in anterior zone 2 and zone 3). 

Further studies would be beneficial to address the ideal anti-VEGF agent, its dosage for ROP treatment, and the optimal follow-up regimen for infants after the use of anti-VEGF agents for ROP.

## Figures and Tables

**Table 1:** Survey questions (n = 14) listed on SurveyMonkey completed by ophthalmologists (n = 54) from developed and developing countries.

Survey Questions	Number of Respondents (n)
1. In which country do you practice?	54
2. How many premature babies do you screen (per annum)?	52
3. How many ROP babies have you treated between 1 January 2012 to 31 March 2022?	51
4. Among all ROP babies treated with anti-VEGF between 1 January 2012 to 31 March 2022, how many were treated with anti-VEGF as first-line therapy?	52
5. Between the period of 1 January 2012 to 31 December 2019, what percentage of ROP babies were treated with anti-VEGF as first-line therapy?	53
6. Between the period of 1 January 2020 to 31 March 2022, what percentage of ROP babies were treated with anti-VEGF as first-line therapy?	53
7. What is your first-line treatment option for: AROP (APROP), Type 1 ROP in Zone 1, Type 1 ROP in posterior Zone 2, Type 1 ROP in anterior Zone 2, Type 1 ROP in Zone 3	53
8. When an anti-VEGF treatment is administered, which agent is used?	50
9. What dose of intravitreal anti-VEGF is administered?	50
10. During the period of 1 January 2012 to 31 December 2019, what percentage of ROP babies required repeat anti-VEGF treatment?	52
11. During the period of 1 January 2020 to 31 March 2022, what percentage of ROP babies required repeat anti-VEGF treatment?	52
12. For ROP babies requiring repeat treatment, which treatment modality was used? A-ROP (APROP), Type 1 ROP in Zone 1, Type 1 ROP in posterior Zone 2, Type 1 ROP in anterior Zone 2, Type 1 ROP in Zone 3.	27
13. How long were ROP babies followed up post anti-VEGF treatment (in weeks post-menstrual age)? * Prior to discharge from active screening.	50
14. What is your follow-up interval (in the outpatient setting) for ROP babies who underwent anti-VEGF treatment? * After discharge from active screening.	50

* Active screening is conducted whilst ROP babies are an inpatient in hospital, during the neonatal period.

**Table 2:** Preferred first-line treatment options for the stages of ROP based on the number of responding clinicians in developed (a) and developing (b) countries.

**(a) Developed Countries (number of respondents (n) = 34)**					
	**A-ROP** n (%)	**Type 1 ROP in Zone 1** n (%)	**Type 1 ROP in posterior Zone 2** n (%)	**Type 1 ROP in anterior Zone 2** n (%)	**Type 1 ROP in Zone 3** n (%)
Anti-VEGF	26 (76.4%)	27 (79.4%)	23 (67.6%)	6 (17.6%)	1 (2.9%)
Combination (Laser & Anti-VEGF)	5 (14.7%)	5 (14.7%)	6 (17.6%)	2 (5.8%)	1 (2.9%)
Laser	3 (8.8%)	2 (5.8%)	5 (14.7%)	26 (76.4%)	28 (82.3%)
Other (Cryotherapy)	0	0	0	0	4 (11.7%)
**(b) Developing Countries (number of respondents (n) = 19)**					
	**A-ROP** n (%)	**Type 1 ROP in Zone 1** n (%)	**Type 1 ROP in posterior Zone 2** n (%)	**Type 1 ROP in anterior Zone 2** n (%)	**Type 1 ROP in Zone 3** n (%)
Anti-VEGF	8 (42.1%)	11 (57.8%)	7 (36.8%)	3 (15.7%)	1 (5.2%)
Combination (Laser & Anti-VEGF)	4 (21.0%)	1 (5.2%)	0	1 (5.2%)	0
Laser	7 (36.8%)	4 (21.0%)	9 (47.3%)	13 (68.4%)	14 (73.6%)
Other (Cryotherapy)	0	3 (15.7%)	3 (15.7%)	2 (10.5%)	4 (21.0%)
